# Abnormal Fractional Amplitude of Low-Frequency Fluctuation as a Potential Imaging Biomarker for First-Episode Major Depressive Disorder: A Resting-State fMRI Study and Support Vector Machine Analysis

**DOI:** 10.3389/fneur.2021.751400

**Published:** 2021-11-29

**Authors:** Yujun Gao, Xi Wang, Zhenying Xiong, Hongwei Ren, Ruoshi Liu, Yafen Wei, Dongbin Li

**Affiliations:** ^1^Department of Psychiatry, Renmin Hospital of Wuhan University, Wuhan, China; ^2^Department of Mental Health, Taihe Hospital, Hubei University of Medicine, Shiyan, China; ^3^Department of Psychiatry, Jiangxia District Mental Hospital, Wuhan, China; ^4^Department of Medical Imaging, Tianyou Hospital Affiliated to Wuhan University of Science and Technology, Wuhan, China; ^5^Department of Neurology, The Fourth Affiliated Hospital of Harbin Medical University, Harbin, China; ^6^First Department of Neurology and Neuroscience Center, Heilongjiang Provincial Hospital, Harbin, China; ^7^Department of Neurology, The First Affiliated Hospital of Harbin Medical University, Harbin, China

**Keywords:** fractional amplitude of low-frequency fluctuation, first-episode major depressive disorder, resting-state fMRI, support vector machine, imaging biomarker

## Abstract

**Objective:** Major depressive disorder (MDD) is a psychiatric disorder with serious negative health outcomes; however, there is no reliable method of diagnosis. This study explored the clinical diagnostic value of the fractional amplitude of low-frequency fluctuation (fALFF) based on the support vector machine (SVM) method for the diagnosis of MDD.

**Methods:** A total of 198 first-episode MDD patients and 234 healthy controls were involved in this study, and all participants underwent resting-state functional magnetic resonance imaging (fMRI) scanning. Imaging data were analyzed with the fALFF and SVM methods.

**Results:** Compared with the healthy controls, the first-episode MDD patients showed higher fALFF in the left mid cingulum, right precuneus, and left superior frontal gyrus (SFG). The increased fALFF in these three brain regions was positively correlated with the executive control reaction time (ECRT), and the increased fALFF in the left mid cingulum and left SFG was positively correlated with the 17-item Hamilton Rating Scale for Depression (HRSD-17) scores. The SVM results showed that increased fALFF in the left mid cingulum, right precuneus, and left SFG exhibited high diagnostic accuracy of 72.92% (315/432), 71.76% (310/432), and 73.84% (319/432), respectively. The highest diagnostic accuracy of 76.39% (330/432) was demonstrated for the combination of increased fALFF in the right precuneus and left SFG, along with a sensitivity of 84.34% (167/198), and a specificity of 70.51% (165/234).

**Conclusion:** Increased fALFF in the left mid cingulum, right precuneus, and left SFG may serve as a neuroimaging marker for first-episode MDD. The use of the increased fALFF in the right precuneus and left SFG in combination showed the best diagnostic value.

## Introduction

Major depressive disorder (MDD) is a severe psychiatric disorder affecting more than 264 million people around the world (1) and is characterized by cognitive and affective dysfunction. Previous studies have revealed that MDD participates in some mechanisms/pathophysiologies, such as genetics, environmental factors, neuroendocrinology, inflammation, neuroplasticity, and monoamines (2). All these molecular and cellular alterations ultimately result in brain network structure and functional network connectivity. Several studies have confirmed that MDD is relevant to specific brain circuits or networks, such as the default mode network (DMN) (3), affective-salience circuit (4), and frontoparietal cognitive control circuit (5). Concomitantly, abnormal brain circuits/networks have also been confirmed to be related to the symptoms of MDD. Frontoparietal network hypoconnectivity was found in MDD and was associated with attention regulation (6). Major depressive disorder is related to activity within the subcallosal cingulate cortex, an extensively connected element of the limbic system that regulates feelings of sadness (7). Major depressive disorder was also associated with decreased connectivity between the ventromedial prefrontal cortex and dorsal striatum, which correlated with motor speed and psychomotor slowing (8).

With the rapid development of neuroimaging technology, particularly resting-state functional magnetic resonance imaging (fMRI), our understanding of MDD mechanisms may move a step further. Due to its non-invasive characteristics, resting-state fMRI has been widely used in the neuropathology of neuropsychiatric disorders, such as autism (9), obsessive-compulsive disorder (10), anxiety disorders (11), and MMD (12). Resting-state fMRI relies on the spontaneous low-frequency fluctuation in the blood oxygen level-dependent signal, which could reflect the spontaneous activity of neurons (13). During resting-state fMRI, subjects were asked to be awake and quiet without conscious mental activity. Compared with task-state fMRI, resting-state fMRI is more convenient for clinical application. Several analysis methods of resting-state fMRI have been widely used in the assessment of disease processes and disease diagnostics, such as regional homogeneity (14), voxel-based morphometry (15), network homogeneity (16), voxel-mirrored homotopic (17), functional connectivity (18), and mean-square successive difference (19). Although most resting-state fMRI studies have confirmed correlations between spatially different brain regions from functional integration, these methods do not intuitively provide information on the amplitude of brain activity in all brain regions. For instance, regional homogeneity is employed mainly in the measurement of the functional coherence of a given voxel with its nearest voxels (20), and functional connectivity is used mainly for measuring connections between brain areas or individual voxels within a network (21, 22).

The fractional amplitude of low-frequency fluctuation (fALFF) is frequently used for the brain function evaluation and can be used to characterize spontaneous brain activity (23). Due to the high temporal stability, fALFF was confirmed to have high potential value as a diagnostic marker in a previous study (24). Compared with amplitude of low-frequency fluctuation (ALFF) analysis (the original approach), fALFF has fewer physiological noise effects (25). Although the number of MDD studies using fALFF is increasing; few studies have reported the combination of fALFF and support vector machine (SVM) methods. The SVM method is a multivariable pattern recognition technology that can be used to analyze the data and detect patterns (26). Meanwhile, the SVM is ideally suited for high-dimensional data, with many more features than the number of samples, which is often true of experiments. An optimal separating hyperplane of the high-dimensional space can be confirmed *via* the SVM, and the samples that are closest to the hyperplane are called the support vector. In the fMRI analysis, a discrimination map can be generated by superimposing the SVM weights back onto the original brain space, so the most significant weights can be visually traced back to the most discriminatory parts of the brain (27). The SVM method has great potential to provide clinically useful criteria for decision-making from such high-dimensional neuroimaging data (28). In this study, we investigated fALFF in first-episode MDD patients, screened brain regions that showed altered fALFF, and discussed their value as potential neuroimaging markers through the SVM method. This study will contribute to the rapid and efficient diagnosis of first-episode MDD.

## Methods

### Subjects

One hundred ninety-eight right-handed first-episode MDD patients were consecutively recruited from the Department of Psychiatry, Tianyou Hospital Affiliated to Wuhan University of Science and Technology. Two hundred thirty-four age-matched (MDD patients: 28.01 ± 7.442, healthy controls: 27.87 ± 6.492) and gender-matched (MDD patients: men/women = 102/96, healthy controls: 130/104) right-handed healthy controls were recruited from those who underwent a standard physical examination at the medical examination center of the Tianyou Hospital Affiliated to Wuhan University of Science and Technology. The diagnosis of depression was based on the Diagnostic and Statistical Manual of Mental Disorders-Fourth Edition (DSM-IV) criteria (29), and all the patients were independently diagnosed by two experienced psychiatrists. Depression severity was assessed by the 17-item Hamilton Rating Scale for Depression (HRSD-17) score, and the patients had a score of more than 18. In the process of clinician-rated depression, the application of HRSD-17 was most prevalent. Based on the HRSD-17, the severity of depression was divided into four categories: <7 scores = normal people, 8–16 scores = mild depression, 17–23 scores = moderate depression, and >24 scores = severe depression. The exclusion criteria for patients were as follows: other Axis I disorders, such as bipolar disorder, acute physical illness, schizophrenia, substance abuse or dependence, substance-induced mood disorder, and a history of head injury resulting in the loss of consciousness. The healthy controls had no history of psychiatric disorders or severe physical illness and no family history of psychiatric disorders. All participants gave written informed consent before the study. Our study was approved by the Medical Ethics Committee of the Tianyou Hospital Affiliated to Wuhan University of Science and Technology and performed in accordance with the Declaration of Helsinki.

### Behavioral Paradigms

The executive control function was assessed by the attentional network test (ANT) (30). The stimulus signals of ANT visually appear on a screen, and the subjects were required to correctly and quickly identify the orientation in which a central target arrow pointed. The reaction time (RT) of all the subjects was recorded, and the executive control reaction time (ECRT) was calculated by subtracting the consistent arrow direction RT from the inconsistent arrow direction RT. A longer ECRT indicated inferior executive control performance [the detailed steps were excerpted from our previous study (31), and detailed information can be found in the Supplementary Material].

### Image Acquisition

All the resting-state MRI data were acquired on an Achieva 3.0T scanner (Philips, Amsterdam, the Netherlands) at the Tianyou Hospital Affiliated to Wuhan University of Science and Technology. All the participants were instructed to lie still and stay awake with their eyes closed. The scanning parameters were as follows: repetition time/echo time (TR/TE) 2,000/30 ms; 31 slices; 90° flip angle; 220 × 220 mm field of view; 5 mm slice thickness; and 1 mm pitch [the detailed steps were excerpted from our previous study (32), and detailed information can be found in the Supplementary Material].

### Data Preprocessing

Imaging data from the resting-state fMRI were preprocessed using the DPARSF software in MATLAB (23). To reduce the influence of participants' adaption time and the instability of the initial signal, the first five-time points were discarded. Slice time and head motion were corrected. None of the participants had more than 2 mm of maximum displacement in the x-, y-, or z-axis, or more than 2° of maximum rotation. The corrected imaging data were spatially normalized to the standard Montreal Neurological Institute space and resampled with 1 × 1 × 1 mm^3^. Then, the obtained images were band-pass filtered (0.01–0.08 Hz) and linearly detrended. Several spurious covariates were removed, such as the signal from a region centered in the white matter and the signal from a ventricular seed-based region of interest, as well as the six head-motion parameters obtained by rigid body correction. The global signal was retained during the processing of the resting-state functional connectivity data [the detailed steps were excerpted from our previous study (32), and detailed information can be found in the Supplementary Material].

### fALFF Analysis

The fALFF analysis was performed with REST software. The fALFF values were calculated according to the previous study (33). The time series for each voxel were transformed to the frequency domain by using fast Fourier transform, and the power spectrum was then computed and square root-transformed at each voxel. The averaged square root was obtained as the ALFF across 0.01–0.08 Hz at each voxel. After that ALFF was calculated, the sum of amplitudes across 0.01–0.08 Hz was divided by that across the entire frequency range, and fALFF was acquired (detailed information can be found in the Supplementary Material).

### Classification Analysis

The SVM method was operated using the LIBSVM software package in MATLAB and was applied to test the ability to differentiate MDD from healthy controls using the extracted fALFF values in abnormal brain regions. The best parameters, C (penalty coefficient), and g (gamma) were selected, and the LIBSVM tool was used to evaluate a grid of parameters. Through the LIBSVM, all the parameter settings' accuracies were acquired, and then, the highest cross-validation accuracy of the parameters was determined (detailed information can be found in the Supplementary Material).

### Statistics

The two-sample *t*-test was used to analyze the age, years of education, HRSD-17, and ECRT between MDD patients and healthy controls, and the Chi-squared test was used to analyze the gender distributions using SPSS 22.0. Age, years of education, and frame-wise displacement all were used as covariates. Spearman correlation was used to detect any correlations between abnormal fALFF and clinical variables. The significance level was set at *p* < 0.05.

To identify the group differences, an analysis of covariance was used on individual whole-brain fALFF maps of the two groups in a voxel-by-voxel manner. The voxel-based mean fALFF values were withdrawn from the abnormal values in brain regions. The results were thresholded at *p* < 0.01, GRF-corrected using cluster-extent based thresholding in REST with primary threshold of *p* < 0.001.

## Results

### Characteristics of the Subjects

A total of 198 first-episode MDD patients and 234 healthy controls were involved in this research. The demographic and clinical data of the participants are provided in Table 1. No significant differences were observed in terms of age, sex, or educational level. However, first-episode MDD patients had a longer ECRT than healthy controls.

**Table 1:** The *p*-value for the gender distribution was obtained by the Chi-square test.

**Characteristics**	**Patients (*n* = 198)**	**HCs (*n* = 234)**	***p*-values**
Gender (men/women)	198 (102/96)	234 (130/104)	0.401
Age, years	28.01 ± 7.442	27.87 ± 6.492	0.832
Years of education, years	12.05 ± 3.325	12.55 ± 2.931	0.100
HRSD-17	23.63 ± 2.547		
ECRT (ms)	141.10 ± 49.400	84.11 ± 52.746	0.000

*The other p-values were obtained by two sample t-tests. HC, healthy controls; ECRT, executive control reaction time; HRSD-17, 17-item Hamilton rating scale for depression*.

### Group Differences in fALFF

**Figure 1:**
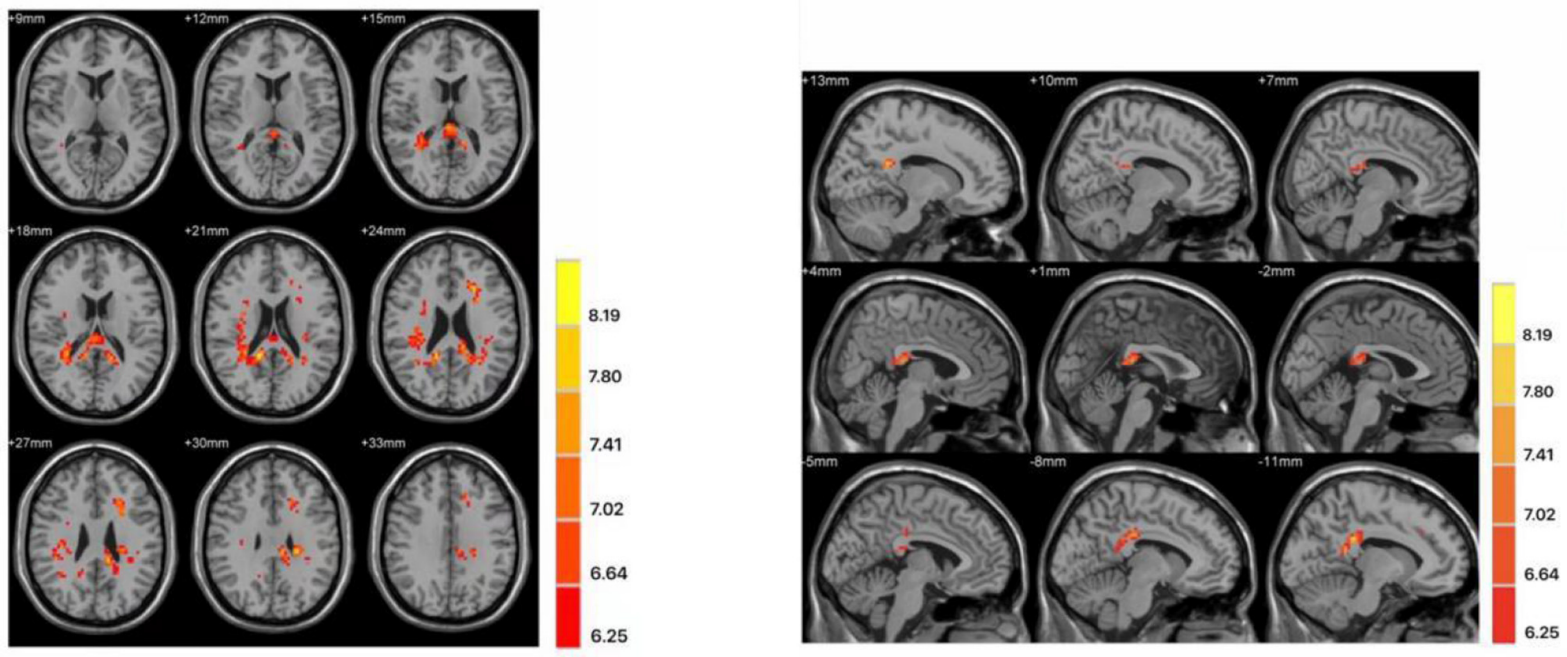
Differences in fractional amplitude of low-frequency fluctuation (fALFF) values between first-episode major depressive disorder patients and healthy controls. Increased fALFF values (left mid cingulum, right precuneus, and left superior frontal gyrus) were presented on the red color, and the color bar indicates the *T* values of the group analysis. Left: transverse plane; Right: sagittal plane.

The two-sample *t*-test was used to test significant differences in fALFF values between first-episode MDD patients and healthy controls. Patients showed significantly higher fALFF in the left mid cingulum, right precuneus, and left superior frontal gyrus (SFG) than healthy controls (Figure 1; Table 2).

**Table 2:** Clusters with abnormal fractional amplitude of low-frequency fluctuation in the patients with major depressive disorder.

**Cluster location**	**Peak (MNI)**			**Number of voxels**	***T*-value**
	***X***	***Y***	***Z***		
Left mid cingulum	−24	−27	30	145	8.0602
Right precuneus	15	−48	21	235	8.5775
Left SFG	−21	24	24	58	8.0042

*MNI, Montreal neurological institute; SFG, superior frontal gyrus*.

### SVM Results

**Figure 2:**
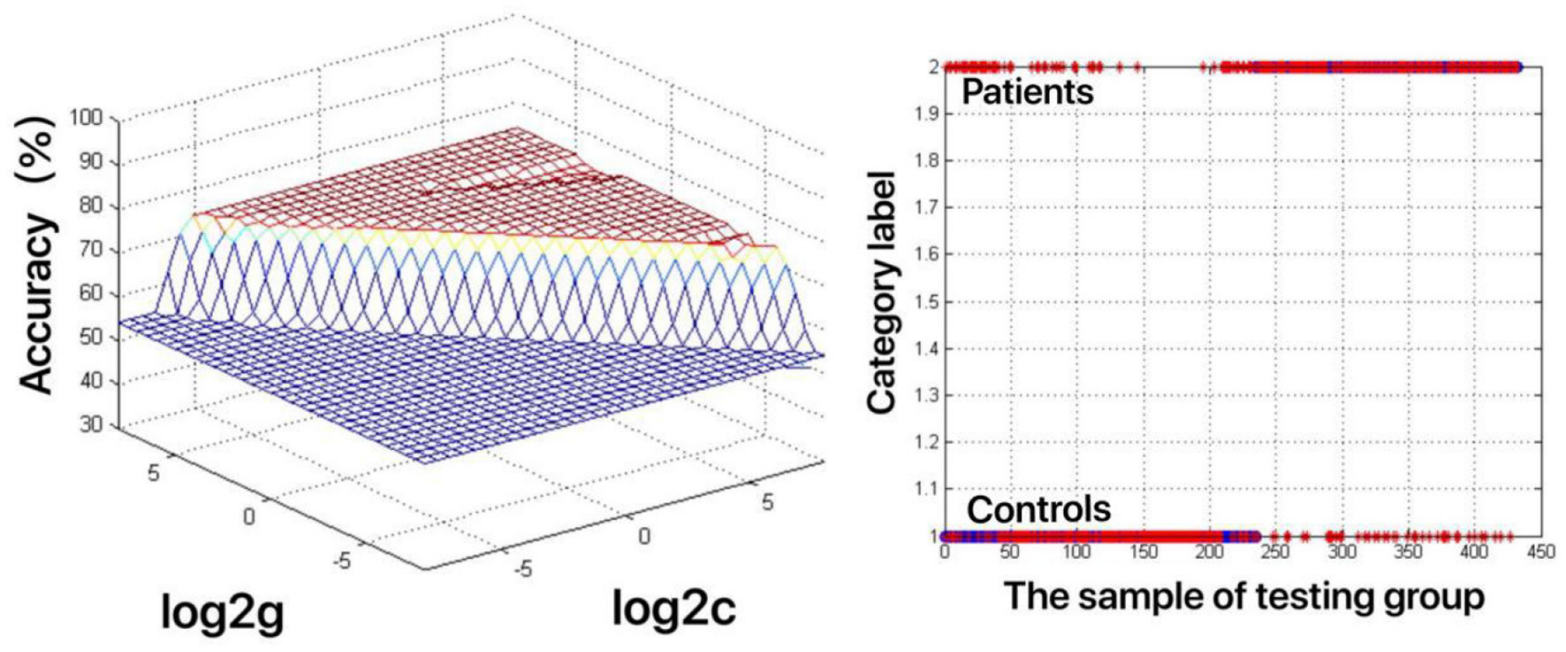
Visualization of classifications through support vector machine (SVM) using the increased fractional amplitude of low-frequency fluctuation (fALFF) values in the left superior frontal gyrus (SFG) to discriminate the MDD patients from healthy controls. Left: SVM parameters result of 3D view. g means gamma, c means penalty coefficient. Right: Classified map of the fALFF values in the left SFG. Blue circle means true value and the red asterisk means predict value.

**Figure 3:**
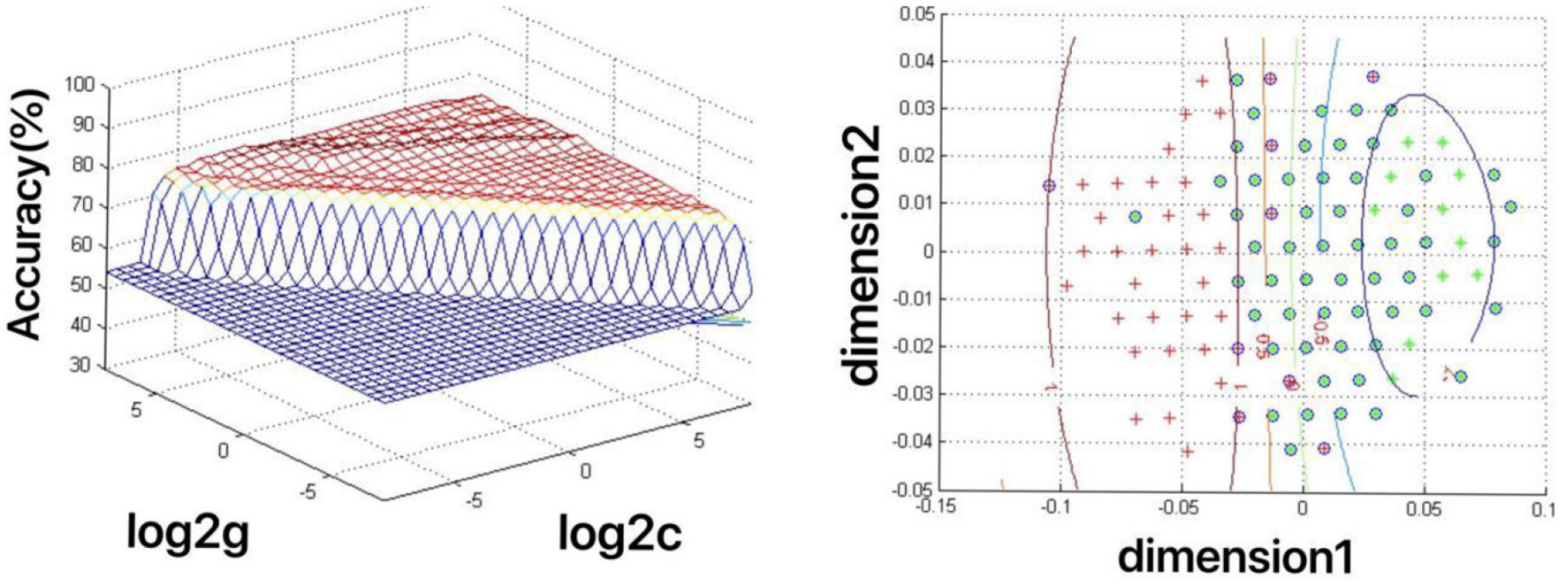
Depiction of classifications based on the support vector machine (SVM) method using a combination of fractional amplitude of low-frequency fluctuation (fALFF) values in the right precuneus and left SFG to differentiate the first-episode major depressive disorder (MDD) patients from the healthy controls. Left: SVM parameters result of 3D view. g means gamma, c means penalty coefficient. Right: dimension 1 and dimension 2 represent the fALFF values in the right precuneus and left SFG, respectively. Green crosses represent the first-episode MDD patients, and the red crosses represent the healthy controls.

The increased fALFF values of these three brain regions (left mid cingulum, right precuneus, and left SFG) in the first-episode MDD patients were analyzed by the SVM method. The classification accuracies were as follows: left mid cingulum = 72.9167% (315/432), right precuneus = 71.7593% (310/432), and left SFG = 73.8426% (319/432). The increased fALFF in the left SFG showed the highest diagnostic accuracy of 73.8426%, with a sensitivity of 81.82% (162/198), and a specificity of 72.65% (170/234) (Figure 2). Meanwhile, the SVM results showed that a combination of increased fALFF in the right precuneus and left SFG exhibited the highest accuracy of 76.3889% (330/432), with a sensitivity of 84.34% (167/198), and a specificity of 70.51% (165/234) (Figure 3).

### Correlation of fALFF With Clinical Variables

**Figure 4:**
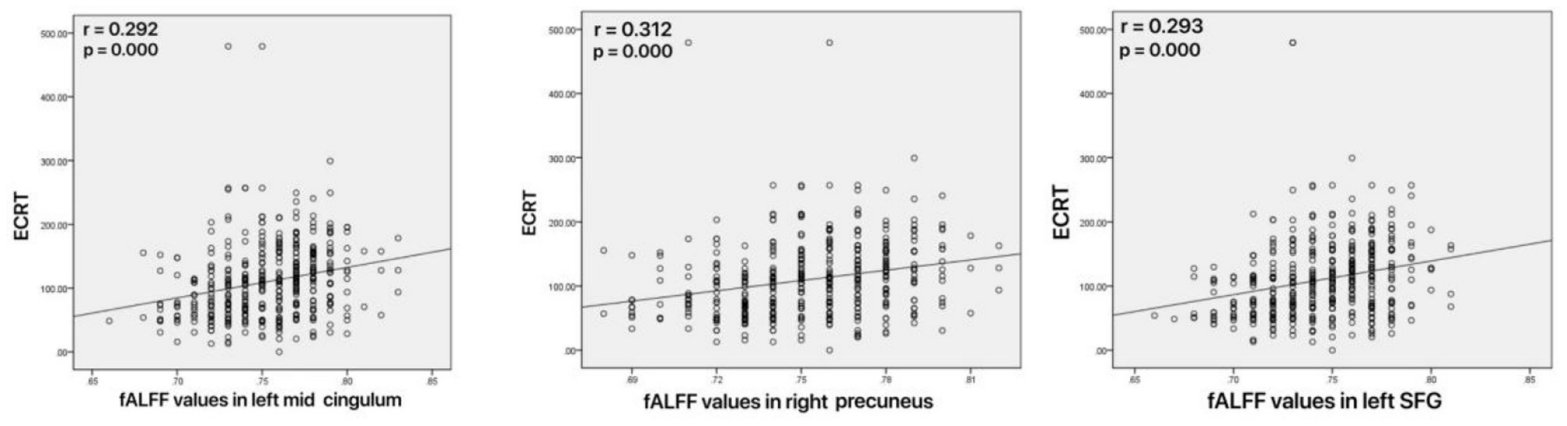
Correlations between abnormal fractional amplitude of low-frequency fluctuation (fALFF) and executive control reaction time (ECRT). Left: Positive correlation between the fALFF values in the left mid cingulum and ECRT; Mid: Positive correlation between the fALFF values in the right precuneus and ECRT; Right: Positive correlation between the fALFF values in the left SFG and ECRT.

**Figure 5:**
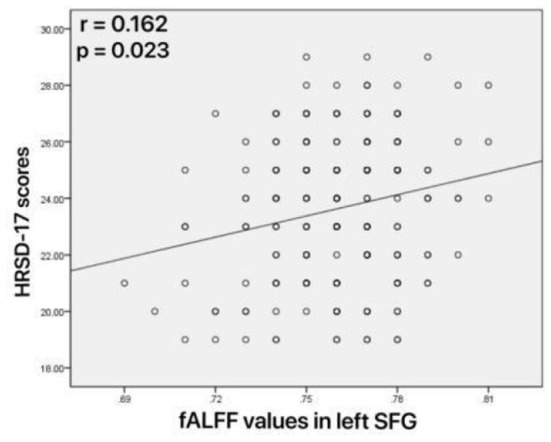
Correlations between abnormal fractional amplitude of low-frequency fluctuation (fALFF) and 17-item Hamilton Rating Scale for Depression (HRSD-17) scores. Positive correlation between the fALFF values in the left superior frontal gyrus and HRSD-17 scores.

As shown in Figure 4, increased fALFF values in the left mid cingulum, right precuneus, and left SFG were positively correlated with ECRT. The increased fALFF values in the left mid cingulum and left SFG were positively correlated with the HRSD-17 scores (Figure 5).

## Discussion

The objective and rapid diagnosis of MDD have always been a hot spot in clinical research; however, there are no objective lab-based diagnostic methods for MDD, and its diagnosis still depends on depression scales and subjective analysis. With the development of brain imaging technology, fMRI research is increasingly used to assist clinical diagnosis. To the best of our knowledge, this study was the first to explore the utility of altered fALFF values in the left mid-cingulum, right precuneus, and left SFG as neuroimaging markers for the first-episode MDD, combined with the SVM method.

The cingulum is a key component of the limbic lobe, and it is a major interconnecting apparatus of all cerebral lobes (34). It has been described as the “seat of dynamic vigilance by which environmental experiences are endowed with an emotional awareness” by Papez (35). Due to its cytoarchitectonic characteristics and the distribution of receptors, the cingulate gyrus is divided into four subregions, such as the anterior cingulate cortex, middle cingulate cortex, posterior cingulate cortex, and retrosplenial cortex (36). The middle cingulate cortex has been implicated in negative affect and cognitive control (37) and is mainly enrolled in the selection of responses according to their motivational relevance (34). Moreover, several memory task studies have shown evidence that the cingulum participates in working memory (38), especially the mid cingulum. Although the memory task was not included in the present study, similar results were noticed. In our study, an ANT was performed to evaluate executive control function, and first-episode MDD patients showed longer ECRT than healthy controls. Consistent with our study, Rao et al. (39) confirmed that the left mid cingulum is closely related to attention networks, which also modulate cognitive-linguistic conflict. Cerebral perfusion single-photon emission computed tomography (40) revealed that the number of depressive episodes was negatively correlated with perfusion of the right anterior cingulum, and the depression duration was negatively correlated with perfusion of the right anterior cingulum. In our study, increased fALFF values of the left mid cingulum were found in the patients with MDD, and the altered fALFF values were positively correlated with ECRT. As mentioned above, a longer ECRT indicated inferior executive control performance, and thus we speculated that abnormal fALFF in the left mid cingulum has a critical role in the executive function of MDD.

The precuneus is one of the highest resting metabolic rates of all brain structures and is located on the posteromedial portion of the parietal lobe. As the precuneus is hidden in the interhemispheric fissure and rarely damaged in brain diseases such as ischemic stroke and accidents, this cortical area has traditionally received little study. The precuneus, as the functional core of the DMN, was one of the most important findings of recent neuroimaging studies (41, 42). However, the precuneus is closely related to the DMN, which is thought to be tightly associated with MDD, we confirmed in our present study that the precuneus is involved in the process of MDD. Hermesdorf et al. (43) found that compared with never-depressed controls, MDD patients showed lower voxel-mirrored homotopic connectivity (VMHC) in the precuneus. In the present study, we observed increased fALFF values in the right precuneus, and the SVM results showed an accuracy of 71.7593% (310/432) to differentiate the first-episode MDD from healthy controls. Although no correlation between fALFF and HRSD-17 scores was found in our study, the increased fALFF of the right precuneus was positively correlated with ECRT.

The SFG is an important functional region located in the superior prefrontal cortex. Based on diffusion tensor tractography, the SFG can be divided into three subregions: the anteromedial SFG, dorsolateral SFG, and posterior SFG (44). Remarkably, both the anteromedial SFG and dorsolateral SFG were mainly correlated with the DMN (44), similar to the precuneus. Meanwhile, the SFG also plays a critical role in working memory, similar to the cingulum (45, 46). From all the descriptions discussed above, it is not difficult to find that there is a tight functional correlation among these three brain regions (precuneus, cingulum, and SFG) in our study. Several studies on the abnormal SFG in MDD have been reported; for example, Xiong et al. (47) confirmed that altered left SFG gyrification means state-independent changes in MDD. Lebedeva et al. (48) found that atrophy in the left SFG developed in parallel with depressive symptoms. In the present study, we observed increased fALFF values in the left SFG, and the SVM results showed an accuracy of 73.8426% (319/432) to differentiate the first-episode MDD from healthy controls. However, a previous study showed the opposite result, which suggested that decreased fALFF in the left SFG may be a trait neuroimaging marker for MDD (49). We hypothesized that the number of samples was the key factor (only 14 MDD patients were enrolled in the previous study). Our results showed increased fALFF values in the left SFG, and the altered fALFF values were positively correlated with the ECRT and HRSD-17 scores. Thus, our data suggested that increased fALFF of the left SFG may contribute to the pathophysiological processing of MDD.

Over recent years, neuroimaging markers have become an intense focus of MDD diagnosis. Many neuroimaging studies have been reported, such as brain volumetric MRI (50), diffusion tensor imaging (51), and single-photon emission computed tomography (52). However, the results of these studies are inconsistent, and several factors, such as different study designs and small sample sizes, may contribute to this inconsistency. In this study, we collected a large sample size and chose the SVM method, which has been widely used for computer-aided diagnosis and prediction in disease diagnosis. Fractional amplitude of low-frequency fluctuation is a common index of resting-state fMRI, which is closely associated with the pathophysiological mechanisms of MDD. By measuring the abnormal fALFF values of MDD and first-degree relatives, Song et al. (53) suggested that fALFF could be utilized as a neuroimaging marker for monitoring MDD progression. Qiu et al. (54) reported that after electroconvulsive therapy, MDD patients showed obviously decreased fALFF. In our study, SVM analysis showed that increased fALFF values in the left mid cingulum, right precuneus, and left SFG could be used to distinguish first-episode MDD patients from healthy controls, and the combination of increased fALFF in the right precuneus and left SFG exhibited the highest accuracy of 76.3889% (330/432), with a sensitivity of 84.34% (167/198), and a specificity of 70.51% (165/234).

Some limitations exist in our study. The influence of physiological noises such as cardiac and respiratory rhythm cannot be completely removed. Another limitation is the sample source as all the subjects were recruited from the China's Hubei province.

## Conclusion

In conclusion, the altered fALFF in the left mid cingulum, right precuneus, and left SFG may be state-related changes of MDD. And, the combination of increased fALFF in the right precuneus and left SFG may be a potential neuroimaging marker for the first-episode MDD.

## Data Availability Statement

The raw data supporting the conclusions of this article will be made available by the authors, without undue reservation.

## Ethics Statement

The studies involving human participants were reviewed and approved by the Medical Ethics Committee of the Tianyou Hospital Affiliated to Wuhan University of Science and Technology. The patients/participants provided their written informed consent to participate in this study. Written informed consent was obtained from the individual(s) for the publication of any potentially identifiable images or data included in this article.

## Author Contributions

DL and RL: writing the article. XW, ZX, and HR: collection and assembly of data. YG and YW: research concept and design, data analysis, and interpretation. All authors contributed to the article and approved the submitted version.

## Funding

The investigation was supported by the grant from the Health Commission of Hubei Province Scientific research project (Grant No. 2020CFB512).

## Conflict of Interest

The authors declare that the research was conducted in the absence of any commercial or financial relationships that could be construed as a potential conflict of interest.

## Publisher's Note

All claims expressed in this article are solely those of the authors and do not necessarily represent those of their affiliated organizations, or those of the publisher, the editors and the reviewers. Any product that may be evaluated in this article, or claim that may be made by its manufacturer, is not guaranteed or endorsed by the publisher.
