# Supplementary material for: Abnormal Fractional Amplitude of Low-Frequency Fluctuation as a Potential Imaging Biomarker for First-Episode Major Depressive Disorder: A Resting-State fMRI Study and Support Vector Machine Analysis
Source: Front Neurol. 2021 Nov 29;12:751400. doi: 10.3389/fneur.2021.751400 (PMC8666416; doi:10.3389/fneur.2021.751400)
Supplement: Supplementary file 1 [file Table_1.DOCX]

***Supplementary Material***

1 Supplementary Methods

**Behavioral Paradigms**

The ANT was devised by Fan and his colleagues. In the center of the screen a “+” was displayed as the fixation point. The stimulus signals could appear above or below the center of the screen in the form of a target (“→”) or a foil (“∗”). The foil could appear in one of the following 4 conditions: no foil, one foil at the
center, one foil above and another one below the center, and one foil either above or below the center. The target could appear in one of the following 3 conditions: a single arrow, 5 arrows in the same direction, and 5 arrows in different directions. The subjects were required to correctly and quickly determine the orientation of the target. The alertness network RT was calculated by subtracting the double-cue RT from the no-cue baseline RT.

**Image Acquisition**

An Achieva 3TMRI scanner (Philips, Netherlands) was utilized for resting-state functional magnetic resonance imaging (rsfMRI). Patients were asked to lie down and close their eyes but remain awake. A prototype quadrature birdcage head coil filled with foam was used to minimize head movement. Functional imaging had the following parameters: ratio of repetition time to echo time (TR/TE) (2,000/30 ms), slice thickness (5 mm), pitch (1 mm), field of view (220 × 220 mm) and flip angle (90◦). On the
structural scan (T1-weighted), the following settings were used: spin-echo sequence, repetition time (TR) = 20 ms, echo time (TE) = 3.5 ms, slice thickness = 1 mm, and field of view (FOV) = 220 × 220 mm.

**Data Preprocessing**

Imaging data of rs-fMRI were preprocessed by using DPARSF software in MATLAB. The first 5 time points were removed. Slice time and head motion were corrected. No participants had more than 2mm of maximal displacement in x, y, or z axis and more than 2° of maximal rotation. The structure of each patient was registered to its functional image. The structure of each patient was divided, and a template was created to normalize the structures of the patients after they were defined according to the Montreal Neurological Institute (MNI) standard template, the standardization process of the spatial deformation of the modulation and the structure of the voxel size using 1 × 1 × 1 mm^3^. Finally, the use of the structure of each patient to the function of the conversion matrix was also standardized to the MNI space. During the process of functional image normalization, head motion parameters, white matter signal, and cerebrospinal fluid signal were used as removal covariates , and voxel size of 3 × 3 × 3 mm^3^ was used as functional covariate. The obtained images were subsequently smoothed with an 8mm full width at half-maximum Gaussian kernel, band pass filtered (0.01–0.1Hz), and linearly detrended to lessen the effect of low-frequency drifts and physiologic highfrequency noise. Several spurious covariates were removed, including signal from a region centered in the white matter, 6 head motion parameters obtained by rigid body correction, and signal from a ventricular ROI. The global signal removal may introduce artifacts into the data and distort resting-state connectivity patterns. Furthermore, the regression of the global signal may significantly distort results when studying clinical populations. Therefore, the global signal was preserved.

**fALFF analysis**

After Data Preprocessing, linear trend was removed. Then the fMRI data were temporally band-pass filtered (0.01 < f < 0.08 Hz) to reduce the very low-frequency drift and highfrequency respiratory and cardiac noise. ALFF analysis was performed using the AFNI software. The time series for each voxel was transformed to the frequency domain and the power spectrum was then obtained. Since the power of a given frequency is proportional to the square of the amplitude of this frequency component, the square root was calculated at each frequency of the power spectrum and the averaged square root was obtained across 0.01–0.08 Hz at each voxel. This averaged square root was taken as the ALFF. In our previous work, the ALFF of each voxel was divided by the individual global mean of ALFF within a brain-mask, which was obtained by removing the tissues outside the brain using Matlab software. In the current work, the individual data was transformed to Z score (i.e., minus the global mean value and then divided by the standard deviation) other than simply being divided by the global mean. Spatial smoothing was conducted on the Z maps with an isotropic Gaussian kernel of 8 mm of full-width at half-maximum. The Z maps were transformed to the Talairach and Tournoux coordinates and one-sided one-sample t-test was performed on the Z maps.

The procedure of data analysis of fALFF was similar to the ALFF mentioned above. After the linear trend was removed, the time series for each voxel were transformed to a frequency domain without band-pass filtering. The square root was calculated at each frequency of the power spectrum. The sum of amplitude across 0.01–0.08 Hz was divided by that across the entire frequency range, i.e., 0–0.25 Hz. Further procedures were the same as the ALFF analysis.

**Classification Analysis**

We use LibSVM method based on Weka. LibSVM is a library about the SVM developed by Professor Lin et al. in 2001. It has been widely used in bioinformatics. It has the advantages of being a small program that is flexible, with less inputting parameters, is open source to expand easily, and thus has become the most widely used SVM Library in China. This library tool can be accessed at https://www.csie.ntu.edu.tw/~cjlin/. Weka is a free and noncommercial mining platform, which has a series of functional modules that basically meet various needs in data analysis, such as a variety of different classification and regression algorithms and performing cross validation during classification, automatically. LibSVM classification has been supported since Weka version 3.5.

In this experiment, the radial basis function (RBF) is adopted as the kernel function, which is also the default setting in LibSVM. Two parameters, the cost (c) and gamma (g), need to be determined before building the classification model by using Weka. The parameter c is called the penalty coefficient. The higher the value of c is, the easier it is to over fit. And g is a parameter of RBF function after it is selected as kernel which affects the speed of process of training and prediction. There is no universally recognized best method for parameter selection, and the common method is to let c and g take values within a certain range, and then set different c and g in the process of training set data classification. Finally, use cross validation to get the classification accuracy verified by the training set in this groups c and g, and select the group with the best classification result by comparison. It is a complicated process, but in LibSVM toolkit, the parameter optimization is automated, and it no longer needs to be manually adjusted. We use the program, grid.py in the LibSVM tool folder to get the optimal parameters.
